# Factors Affecting Circulating Phytosterol Levels: Toward an Integrated Understanding of Atherogenicity and Atheroprotection by Dietary and Circulating Phytosterols

**DOI:** 10.1007/s11883-025-01334-7

**Published:** 2025-10-21

**Authors:** Takanari Nakano, Erina Takashima, Liqing Yu

**Affiliations:** 1 Department of Biochemistry, Faculty of Medicine, Saitama Medical University, 38 Morohongo, Moroyama, Iruma-gun, Saitama, 350-0495 Japan; 2 Division of Endocrinology, Diabetes, and Nutrition, Department of Medicine, University of Maryland School of Medicine, Baltimore, MD USA

**Keywords:** Phytosterols, Sitosterolemia, Cardiovascular risk, Atherogenicity

## Abstract

**Purpose of Review:**

Dysfunction of the ATP-binding cassette G5/G8 heterodimier (ABCG5/G8) leads to sitosterolemia, a condition in which premature atherosclerosis is often observed, thereby linking elevated circulating phytosterols to atherogenicity. Conversely, consumption of phytosterols reduces circulating cholesterol levels and, in animal studies, is anti-atherogenic. The debate over phytosterols’ benefits vs. harms continues without consensus. Two key issues remain: despite extensive research, the etiology of premature atherosclerosis in sitosterolemia is still uncertain, and discussion of phytosterol atherogenicity has not been grounded in quantitative evidence, hindering true risk assessment.

**Recent Findings:**

In this review, we conducted a meta-analysis of circulating cholesterol and phytosterol levels prior to medical interventions in individuals with sitosterolemia. The analysis revealed that severe hypercholesterolemia manifests in the first decade of life but declines rapidly into adulthood, suggesting the presence of hypercholesterolemia-induced atherosclerotic cardiovascular disease (ASCVD). Although ABCG5/G8-deficient animal models recapitulate the symptoms of sitosterolemia, including hematologic abnormalities and organ dysfunction, increased atherogenicity has not been observed in these models. By contrast, the consumption of phytosterol-supplemented foods minimally influences circulating phytosterol levels in the general population and lowers circulating cholesterol levels by approximately 10%. Mendelian randomization studies have indicated an association between circulating phytosterol levels and ASCVD risk; however, genetic background, sterol absorption efficiency, and metabolic disturbances modulate these levels, potentially confounding the interpretation of such associations.

**Summary:**

This review reframes the phytosterol atherogenicity debate through quantitative assessment and clarifies longstanding uncertainties about phytosterol safety, thus contributing to evidence-based risk evaluation and supporting informed clinical and dietary recommendations.

**Supplementary Information:**

The online version contains supplementary material available at 10.1007/s11883-025-01334-7.

## Introduction

Land plants contain non-cholesterol sterol analogs, plant sterols. These compounds are chemically analogous to cholesterol, differing only in the length of their side chains [1]. The daily intake of plant sterols and their saturated forms, stanols, ranges from 200 to 400 mg [2, 3], the amount of which is comparable to that of cholesterol.

**Fig. 1:**
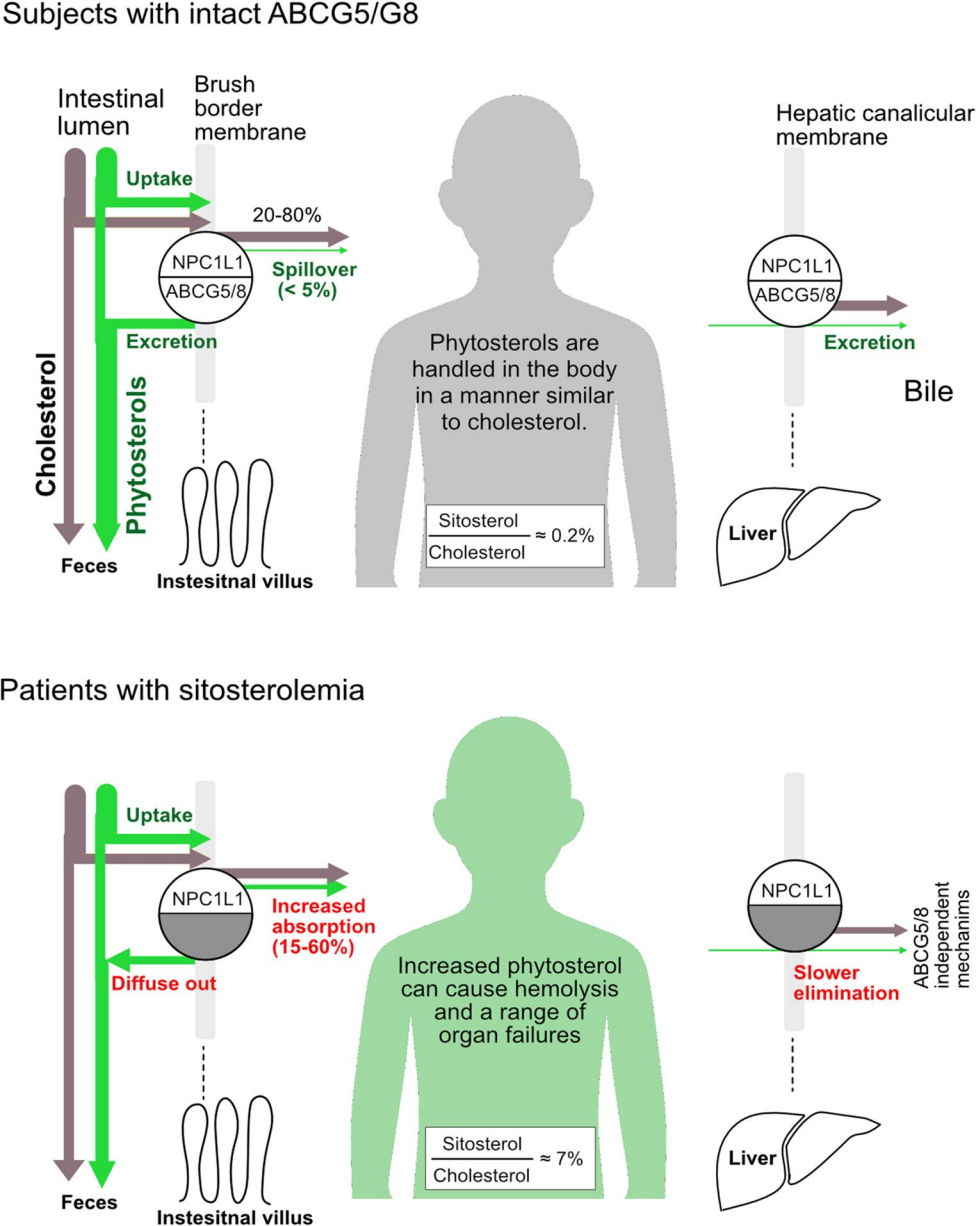
Phytosterol homoeostasis in humans. In subjects with functionally intact ABCG5/G8 (***upper panel***), phytosterol absorption is limited. Absorbed phytosterols are esterified and handled in a manner similar to cholesterol. Hepatic ABCG5/G8 preferentially excretes phytosterols, thereby maintaining low circulating phytosterol levels (approximately 0.2% sitosterol-to-cholesterol ratio; typically, 0.01 mmol/L sitosterol vs. 5 mmol/L cholesterol). ABCG5/G8 and NPC1L1 act together to regulate sterol flux at both membranes [13, 14, 16]. In patients with sitosterolemia (***lower panel***), phytosterol absorption is markedly increased, with sitosterol absorption efficiency reaching 15–60% [5, 17]. The elimination is impaired; consequently, phytosterols accumulate throughout the body. In these patients, the median sitosterol-to-cholesterol ratio is approximately 7%. See Supplementary Tables 1 and the references therein for the data used in this calculation. In the circulation, phytosterols are almost equally esterified. In cells, ABCA1 also effluxes phytosterols, but with lower efficiency.

Plant sterols and stanols, hereafter referred to as phytosterols, are absorbed in the small intestine in a manner similar to that of cholesterol (Fig. 1). However, their absorption efficiency is markedly lower. The absorption efficiency of a given phytosterol species ranges from approximately 0.5 to 5%, whereas roughly half of cholesterol is absorbed [4]. This reduced absorption is primarily attributed to the action of ATP-binding cassette (ABC) transporters G5 and ABCG8 [5–8], the innate eliminators of non-nutritional sterols in the gut [9]. ABCG5 and ABCG8 are half transporters that form a functional sterol efflux transporter heterodimer [10]. ABCG5/G8 is expressed in the intestinal brush border membrane and hepatic canalicular membrane [11, 12], facilitating the expulsion of phytosterols from the small intestine and liver, respectively [7, 8, 10, 13–15].

Loss-of-function mutations in either *ABCG5*, *ABCG8*, or both result in impaired excretion of phytosterols, leading to sitosterolemia, a rare autosomal recessive genetic disease [5]. Patients with sitosterolemia exhibit elevated circulating levels of sitosterol and other phytosterols that are more than 10 to 1,000 times higher (> 0.1 mmol/L for sitosterol) than those in individuals with intact ABCG5/G8 [18].

Once entered, phytosterols behave similarly to cholesterol in the body (Fig. 1). In fact, esterification enzymes, acyl-CoA: cholesterol acyltransferases (ACAT) and lecithin–cholesterol acyltransferase (LCAT), major determinants of sterol metabolism, do not discriminate phytosterols from cholesterol [19, 20], and phytosterols are distributed within tissues in proportionally similar phytosterol-to-cholesterol ratio as in plasma [21]. Phytosterols may tend to be retained within cells for a longer duration because they are less efficient substrates for ABCA1-mediated efflux than cholesterol [22].

Sitosterolemic patients manifest early-onset atherosclerotic cardiovascular disease (ASCVD) [23], raising the concern that elevated circulating phytosterols may accelerate atherosclerosis [24]. In the absence of clear evidence, the potential cardiovascular risk with *normo-phytosterolemia* (< 0.02 mmol/L circulating sitosterol level) remains a subject of ongoing debate. Mendelian randomization studies conducted in populations from Iceland, Denmark, and the UK Biobank have demonstrated an association between circulating phytosterols and cardiovascular risk [25, 26]. Thus, caution has been advised regarding the intake of phytosterol-supplemented foods [27].

The aim of this review is to provide a quantitative overview of the effects of circulating phytosterols on ASCVD, as circulating phytosterol levels in patients, individuals consuming phytosterol-supplemented foods, and animal models have not been systematically compared in evaluating the risk. By integrating evidence from clinical observations, genetic studies, and dietary interventions, this review critically evaluates the relationship between circulating phytosterol levels and cardiovascular risk, and potential confounding factors on this relationship. Overall, there is a lack of sufficient evidence in support of the prevailing hypothesis of circulating phytosterols’ atherogenicity. In this review, we also present the results of a meta-analysis demonstrating that the promotion of atherosclerosis observed in sitosterolemia—which triggered concerns about the atherogenicity of phytosterols— may be largely attributable to childhood severe hypercholesterolemia.

## Factors Affecting Circulating Phytosterol Levels

### Genetics

**Fig. 2:**
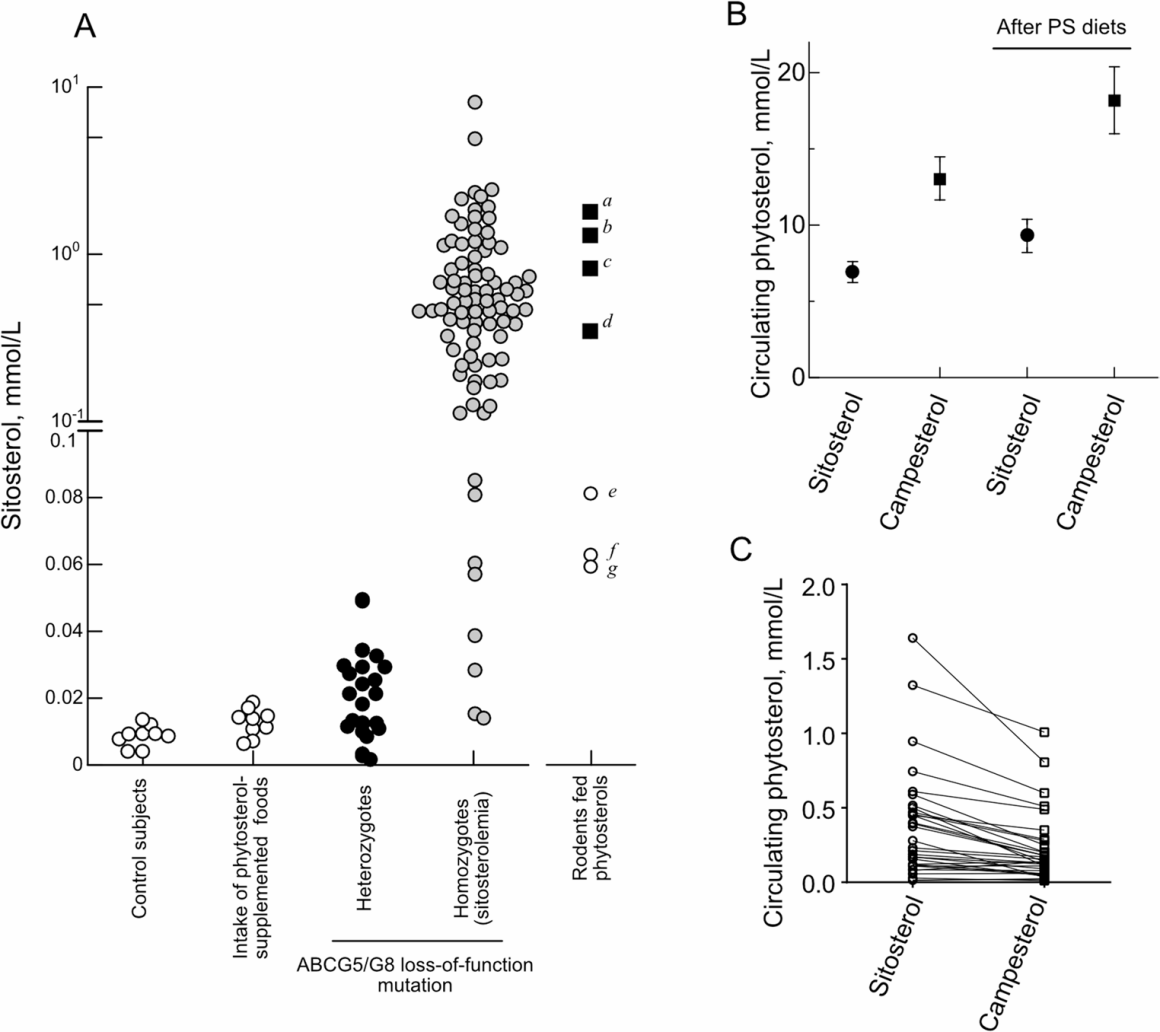
Circulating phytosterol levels. ***A,*** Each plot in control subjects or dietary phytosterol supplementation group represents the mean obtained from respective cohort described in Kritchvsky et al. [29]. The plots in *heterozygotes *group show individual circulating sitosterol levels obtained from subjects carrying monoallelic mutation in *ABCG5/G8* [30–38]. The sitosterol levels in the *homozygous* individuals were obtained at pretreatment. *Rodents fed phytosterols*: *a* and *b*, *Abcg5/g8*-knockout mice fed standard chow and chow supplemented with phytosterols, respectively [39]; *c*, *Abcg5-*deficient WKY rats [40]; *d*, *Abcg5-*deficient stroke-prone spontaneously hypertensive rats [41]; *e*, *Ldlr*-knockout mice; *f* and *g*, *Apoe*-knockout mice fed standard chow or chow supplemented with phytosterols, respectively [42]; *h* and *i*, C57BL/6 mice fed standard chow [42, 43]. ***B,*** Circulating sitosterol and campesterol levels before and after consumption of phytosterol-supplemented foods. The data were obtained from the meta-analysis of randomized controlled trials [44]. Bars show 95% confidence intervals. ***C,*** Circulating sitosterol and campesterol levels in patients with sitosterolemia at pretreatment. Lines connect paired data points from the same individual. In contrast to panel B, sitosterol levels were higher than those of campesterol. For data used in *homozygotes* of *A* and in *C*, see Supplementary Table 1

The heritability of circulating phytosterol concentrations has been recognized since the early stage of genetic research [28]. The most critical genetic determinants of circulating phytosterol levels are *ABCG5* and *ABCG8*. Notably, the function of ABCG5/G8 is obligate for maintaining phytosterol homeostasis in mammals. The normal reference level of sitosterol is in the micromolar range (Fig. 2A).

Biallelic or diallelic loss-of-function mutations in human *ABCG5/G8* directly impair the efficient elimination at the intestinal brush border membrane and reduce phytosterol excretion capacity in the liver, causing sitosterolemia [5, 6] (Fig. 1). The circulating sitosterol level in patients with sitosterolemia may reach 0.65 ± 0.56 mmol/L (mean ± standard deviation) — more than 80-fold higher than the mean levels observed in controls (0.008 ± 0.003 mmol/L) [29].

Subjects with a monoallelic loss-of-function in either *ABCG5* or *ABCG8*, or the heterozygous carriers, exhibit approximately a one-third reduction in the elimination of sitosterol from the body [33]. Circulating phytosterol levels in these carriers range from normal to approximately only 2.5 times higher than the reference (Fig. 2A), a pattern consistent with an autosomal recessive trait of ABCG5/G8 mutations. In a case of sitosterolemia, liver transplantation normalized the circulating phytosterol levels, further demonstrating that a portion of ABCG5/G8 can efficiently remove phytosterols from the body [15].

The absorption process and kinetics of phytosterols in the body are similar to those of cholesterol. In addition to *ABCG5*/*G8*, genome-wide association studies (GWAS) have identified key molecules, such as *HMGCR* (3-Hydroxy-3-methylglutaryl-CoA reductase), *NPC1L1* (Niemann-Pick C1-like 1), and *APOE* (apolipoprotein E), that influence circulating levels of both cholesterol and phytosterols in humans [25, 45, 46]. Although phytosterols are absorbed at a much lower efficiency relative to cholesterol [4], they appear to enter the body primarily via NPC1L1. Ezetimibe treatment significantly reduces circulating phytosterol levels in humans, and *Npc1l1* deficiency in *Abcg5/g8*-knockout mice as well [43, 47–50]. Mice lacking NPC1L1 also likely absorb a lesser amount of phytosterols than wild-type controls, reducing plasma sitosterol levels by approximately 90% or undetectable [43, 51]. The impact of the other gene variants on circulating phytosterol levels in subjects with intact *ABCG5/G8* is minor. For instance, individuals carrying the ApoE ε4 allele tend to exhibit a hyper-absorptive phenotype for sterols, with circulating phytosterol levels elevated by only a few percentage points [52, 53].

### Intestinal Cholesterol Absorption

The efficiency of intestinal cholesterol absorption varies significantly among individuals, ranging from approximately 20–80% [54, 55]. This variability is influenced by genetic factors, drug treatment [56], and metabolic disorders [57, 58]. 

Cholestanol, or 5α-cholestan-3β-ol, is a byproduct of cholesterol metabolites [59]. Cholestanol presents in the circulation at 0.4-1% of cholesterol content [60]. This endogenous cholesterol analog is produced in the liver, excreted in the bile, and absorbed from the small intestine in a manner similar to cholesterol, with approximately one-third of the efficiency compared to cholesterol [60]. Due to its limited endogenous production and the similar enterohepatic circulation kinetics to cholesterol, cholestanol serves as a surrogate marker for cholesterol absorption efficiency. An increase in fractional cholesterol absorption is correlated with an increase in circulating cholestanol levels (*r* > 0.32) [61].

Circulating phytosterol levels correlate with cholesterol absorption efficiency, as both utilize the same absorption pathway. Indeed, the correlations of circulating sitosterol and campesterol with fractional cholesterol absorption efficiency is comparable to that observed for cholestanol (*r* > 0.30 for sitosterol, *r* > 0.42 for campesterol [62]; see also [63]).

**Fig. 3:**
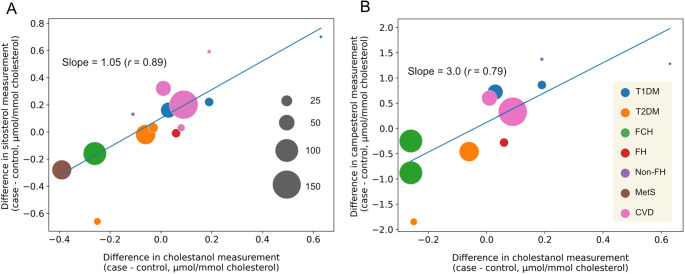
Correlation between circulating levels of phytosterols and cholestanol. The difference in normalized cholestanol levels between cases and controls in clinical studies, shown as each plot, is positively associated with the difference in sitosterol (***A***) and campesterol (***B***). The data were obtained from a systematic review [58]. The slopes and correlation coefficients were obtained by weighting the sample size for each plot. The size of gray circles is proportional to sample size for each study. Cholesterol levels were used to normalize cholestanol, sitosterol, and campesterol values. T1DM, type-1 diabetes mellitus; T2DM, type-2 diabetes mellitus; FCH, familial combined hypercholesterolemia; FH, familial hypercholesterolemia; non-FH, non-familial hypercholesterolemia; MetS, metabolic syndrome; CVD, cardiovascular disease

Mashnafi et al. [58] compiled human case-control studies on metabolic diseases, such as diabetes and hypercholesterolemia, that measured phytosterols and cholesterol absorption markers. We further re-analyzed their data to demonstrate the association of phytosterols with cholesterol absorption efficiency using cholestanol as a marker. The plots showed that the difference in cholestanol and phytosterols between the cases and the controls is strongly correlated (Fig. 3), consistent with the earlier observations [62, 63].

### Cholesterol Homeostasis

**Fig. 4:**
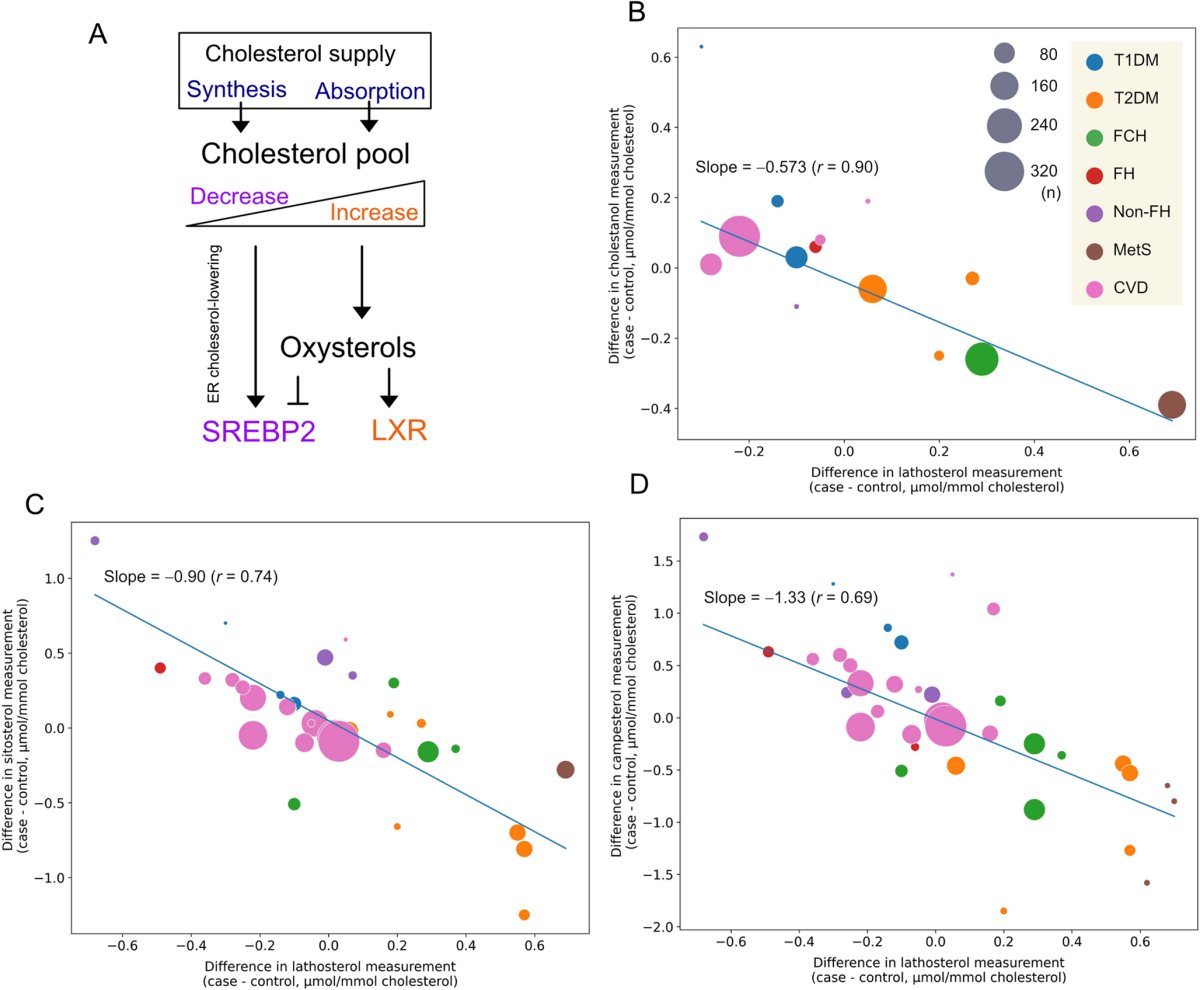
Cholesterol homeostasis regulated by absorption and *de novo* synthesis. ***A***, A homeostatic mechanism of cholesterol abundance in the body: SREBP2 increases the gene expression of *HMGCR*,* LDLR*,* NPC1L1*; LXR increases *ABCA1*,* ABCG1*,* ABCG5/G8.* Oxysterols, whose production correlates with cholesterol abundance, inhibit the activation of SREBP2 and function as agonists for LXR when present in excess [65]. ***B*-*D***, The difference in normalized lathosterol levels between cases and controls in clinical studies, shown as each plot, is negatively associated with those in cholestanol (***B***), sitosterol (***C***), and campesterol (***D***). The slopes and correlation coefficients were obtained by weighting the sample size for each plot. The size of gray circles is proportional to sample size for each study. Cholesterol levels were used to normalize cholestanol, sitosterol, and campesterol values. T1DM, type-1 diabetes mellitus; T2DM, type-2 diabetes mellitus; FCH, familial combined hypercholesterolemia; FH, familial hypercholesterolemia; non-FH, non-familial hypercholesterolemia; MetS, metabolic syndrome; CVD, cardiovascular disease; ER, endoplasmic reticulum

Cholesterol is an essential component of cell membranes, but excess cholesterol can be harmful to the body. Levels of body cholesterol are tightly regulated through a complex interplay of synthesis and absorption. As shown in Fig. 4A, when cellular cholesterol level is low, the membrane-bound transcription factor Sterol Regulatory Element-Binding Protein (SREBP) 2 is activated, releasing its N-terminal domain that enters the nucleus to turn on cholesterol synthesis [64]. When cellular cholesterol level is high, SREBP2 remains in the endoplasmic reticulum, preventing further synthesis of cholesterol. Cholesterol can be oxidized to produce oxysterols which are endogenous agonists for the nuclear factor Liver-X-Receptor (LXR) [65–67]. Activation of LXR signaling limits cholesterol absorption and stimulates the excretion by regulating expression levels of *CYP7A1*, *ABCA1*, *ABCG5/G8*, and *NPC1L1* [68–71].

In clinical settings, *de novo* cholesterol synthesis is often estimated by measuring circulating levels of lathosterol and desmosterol, both of which are precursors of cholesterol. The levels of these two synthesis markers correlate with the estimated rate of cholesterol synthesis (*r* > 0.4 [62]). In another study, even stronger correlations were observed between circulating lathosterol levels normalized by cholesterol abundance and the estimated cholesterol synthesis rate (*r* > 0.7 [56]).

Following the approach used in Fig. 3, we analyzed case-control studies that measured lathosterol, cholestanol, and phytosterols, as listed in the reference [58]. The plots showed that the differences in the sterol species between the cases and the controls were inversely correlated (Fig. 4B-D), confirming that circulating levels of phytosterols, the markers for cholesterol absorption, are tightly linked to lathosterol levels.

### Phytosterol Composition in Diet and Circulation

Sitosterol (24α-ethylcholesterol), campesterol (24α-methylcholesterol), and stigmasterol are the major phytosterol species in the diet, accounting for approximately 60–70%, 15–20%, and 10%, respectively [72]. The order of hydrophobicity is stigmasterol > sitosterol > campesterol, as stigmasterol contains a double bond at C-22 and an additional ethyl group at C-24.

Campesterol has a higher absorption rate (approximately 10%) than the others (about < 5% for sitosterol and stigmasterol) [73], and a lower elimination rate from the liver compared to sitosterol [9, 74]. This is likely because ABCG5/G8 shows a higher preference for effluxing hydrophobic phytosterol species [9, 13, 14]. As a result, the circulating level of campesterol is predominantly higher than that of sitosterol [44]. Intake of phytosterol-supplemented foods increases sitosterol and campesterol levels by an average of 46% and 60%, respectively [29] (Fig. 2B).

In patients with sitosterolemia, sitosterol becomes the dominant phytosterol species in the circulation [75–78]. In the absence of active ABCG5/G8, sitosterol would accumulate to higher levels in the body than campesterol due to its hydrophobicity (0.26 and 0.14 mmol/L of sitosterol and campesterol, respectively, median values; Fig. 2C) [9]. Similarly, in *Abcg5*/*g8*-knockout mice, circulating phytosterol species levels were consistent with dietary composition (sitosterol > campesterol > stigmasterol) [9, 39].

### Phytosterol-Supplemented Diets

Wild-type C57BL/6 mice have circulating levels of sitosterol and campesterol at less than 0.04 and 0.07 mmol/L, respectively [39, 42, 43] (Table 1). Phytosterol-supplemented diets increased circulating sitosterol by approximately 3-fold in wild-type mice [42, 79]. In *Apoe*^−/−^ or *Ldlr*^−/−^ mice, the increase was 7- to 8-fold [42, 84]. These data indicate that mice exhibit higher circulating phytosterol concentrations than humans and demonstrate greater susceptibility to phytosterol-supplemented diets.

**Table 1:** Circulating phytosterol levels in experimental rodents

Species					Chol	Sito	Camp	Stigma	Stanol	
Strain	Characteristics	Diet	Added to diets	Major adverse effects	mmol/L in circulation					References
Rat										
WKY	*Abcg5* deficient	STD chow	0.2% PS	Increased blood pressure	2.09	0.81*				Chen et al. (2010) [40]
WKY	*Abcg5* deficient	STD chow	0.2% Stanols	Increased blood pressure	1.78				0.35**	
SHRSP	*Abcg5* deficient	10% soybean oil			1.18	0.11	0.11			Ratnayake et al. (2000) [41]
SHRSP	*Abcg5* deficient	10% soybean oil	PS-enriched	Shortened lifespan	1.43	0.34	0.50			
Mouse										
C57BL/6	*Apoe* ^−/−^	STD chow			10.0	0.06	0.31			Weingärtner et al. (2008) [42]
C57BL/6	*Apoe* ^−/−^	STD chow	2% PS	Ischemic brain injury	5.0	0.06	0.29			
C57BL/6	Wild-type	STD chow			2.58	0.04	0.06			
C57BL/6	*Abcg5* ^−/−^ */g8* ^−/−^	STD chow†			1.55	1.78	0.65	0.03		Tao et al. (2019) [39]
C57BL/6	*Abcg5* ^−/−^ */g8* ^−/−^	STD chow†	0.2% Sito, 0.2% Stigma	Cardiac injury	1.86	1.28	0.48	0.22		
C57BL/6	Wild-type	STD chow†			3.61	ND	0.07			
C57BL/6	*Apoe* ^−/−^	Western-type diet			24.3		0.01			Weingärtner et al. (2011) [80]
C57BL/6	*Apoe* ^−/−^	Western-type diet	3.4% PS	Endothelial dysfunction	13.7		0.32			
C57BL/6	*Ldlr* ^−/−^	High-fat diet			16.1	0.01	0.04			Bombo et al. (2013) [81]
C57BL/6	*Ldlr* ^−/−^	High-fat diet	2% PS		11.7	0.08	0.09			
C57BL/6	Wild-type	STD chow			3.23	0.01	0.03			Tang et al. (2009) [43]
C57BL/6	*Abcg5* ^−/−^ */g8* ^−/−^	STD chow			0.9	0.53	0.22			Wang et al. (2014) [82]
C57BL/6	Wild-type	STD chow			1.7	< 0.05	< 0.05			

*, as total phytosterols; **, as total phytostanols; †, soy free chow. *PS *phytosterols, *Sito *sitosterol, *Camp *campesterol, *Stigma *stigmasterol, *Stanol *sitostanol, *STD* standard, *WKY* Wistar Kyoto rat (strain, WKY/NCrlCrlj), *SHRSP *Stroke-Prone Spontaneously Hypertensive Rat. For further information regarding the deficiency in *Abcg5*, see Ref. [83]

In humans, the amount of dietary phytosterol intake has a minimal effect on circulating phytosterol levels (Fig. 2A). Total circulating phytosterol remains below 1% of the total sterols even with phytosterol-supplemented foods [44]. The expression of human ABCG5 and ABCG8 in the liver is comparable to, or exceeds, that observed in the small intestine [5]. In contrast, rats and mice show substantially lower hepatic expression of these transporters relative to the small intestinal levels [7, 85]. Consequently, circulating phytosterols may be eliminated more slowly in these species compared to humans, potentially accounting for the observed differences in the susceptibility to phytosterol-supplemented diets. Alternatively, these differences may reflect the quantities of phytosterol administered to human subjects (e.g., 2 g per day) versus experimental animals (e.g., 2% phytosterols by weight in chow).

## Pathogenicity of Phytosterols

Circulating sitosterol levels in patients with sitosterolemia approximate 7% of those of cholesterol (Fig. 1). Owing to their physicochemical similarities to cholesterol, phytosterols are incorporated into cell membranes throughout various tissues. This substitution of cholesterol by phytosterols may lead to hematologic abnormalities and a range of organ dysfunction in humans [9, 21, 86, 87].

The pathogenicity of phytosterols has also been examined in animal models using two main approaches: *Abcg5/g8*-deficient sitosterolemic animals, and animals with intact *Abcg5/g8* fed phytosterol-supplemented diets.

### In *Abcg5/g8*-Deficient Rodents

Studies have shown that *Abcg5/g8-*deficient mice have comparable circulating sitosterol levels (Fig. 2A; Table 1) and defects, such as various hematologic abnormalities and a range of organ dysfunction [39, 88–91], to those observed in patients with sitosterolemia. Reduction of cholesterol synthesis was observed in *Abcg5/g8-*deficient mice as well [9]. Moreover, stigmasterol-supplemented diets induced cardiac injury and fibrosis [39].

Excess phytosterol intake increased blood pressure in *Abcg5*-deficient WKY rats [40, 83] and shortened life span in *Abcg5*-deficient stroke-prone spontaneously hypertensive rats [41].

### In Rodents with Intact *Abcg5/g8*

When phytosterol-supplemented diets were administered to mice and hamsters, atherosclerotic lesion was reduced, as will be discussed in detail below. Overall, rodents with intact *Abcg5/g8* generally tolerate phytosterol supplementation, as do humans (Fig. 2A). Weingärtner et al. [42] reported an exception: vascular dysfunction in *Apoe*^−/−^ mice fed phytosterol-supplemented diets at near-physiological sitosterol levels (0.043 mmol/L, Table 1).

## Phytosterols and Atherosclerosis

To evaluate the atherogenicity of phytosterols, it is essential to consider at least the following three aspects: (1) circulating phytosterol levels, (2) experimental recapitulation of phytosterol-induced atherogenic progression, and (3) known atherogenic factors that cause concomitant increases in phytosterol levels. In this section, we examine these three aspects and then, drawing on the evidence reviewed throughout this article, offer an evidence-based synthesis of the potential benefits and risks of dietary phytosterol intake in the context of ASCVD.

### Circulating Phytosterol Levels

As shown in Fig. 2A, there is a significant difference in the circulating phytosterol levels depending on the functionality of ABCG5/G8, ranging from ‘*hyper-phytosterolemic*’ characterized by circulating sitosterol levels exceeding 0.1 mmol/L in patients with sitosterolemia to ‘*normo-phytosterolemic*’ with levels not exceeding 0.02 mmol/L in individuals with functional ABCG5/G8. Since the pathophysiolgical effects of phytosterols are strongly influenced by their circulating levels, *hyper*- and *normo-phytosterolemic* states warrant distinct consideration.

#### In Patients with Sitosterolemia

Even with ‘*hyper-phytosterolemic*’ states, premature atherosclerosis does not invariably develop in patients with sitosterolemia [92, 93]; this clinical heterogeneity suggests that other factors contribute significantly to the pathogenesis of atherosclerosis in this condition as discussed in detail below.

#### In Individuals with Normo-Phytosterolemia

The difference in circulating phytosterol levels among *normo-phytosterolemic* individuals is attributable to genetic variations of key molecules involved in cholesterol kinetics, cholesterol absorption efficiency, diet, and other factors as described above. A meta-analysis by Genser et al. [94] did not reveal any evidence of an association between circulating concentrations of phytosterols and the risk of ASCVD. Nevertheless, as noted by a German expert panel, it should be challenging to clinically validate the lifelong consequences of increased circulating phytosterol levels, even when these levels remain within the normal range [95].

### Experimental Recapitulation of Phytosterol-Induced Atherogenic Progression

#### In *Abcg5/g8*-Knockout Mice

Circulating phytosterol levels in *Abcg5/g8*-knockout mice are comparable to those observed in patients with sitosterolemia (Fig. 2A; Table 1) [8, 90]. Despite this, Wilund et al. [96] did not observe accelerated atherosclerosis in these animals on the *Ldlr-*knockout background.

#### With Phytosterol-Supplemented Diets

**Fig. 5:**
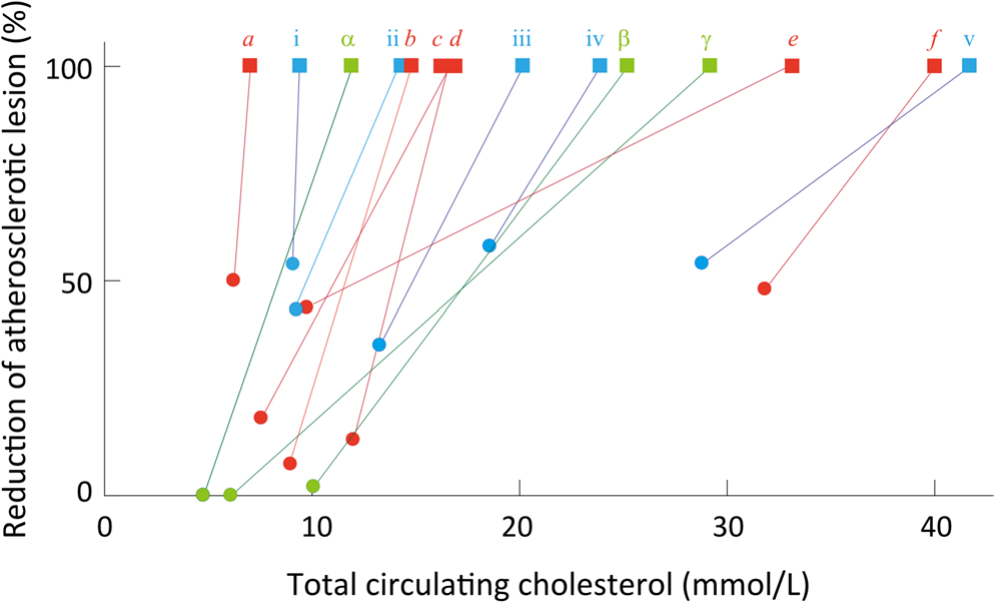
Attenuation of atherosclerotic development in experimental rodents. *Square* and *circle* represent data from control and experimental groups, respectively, and the Y-axis shows the percentage of atherosclerotic lesion scores relative to the controls. *Red symbols* represent rodents fed phytosterols (*a* [97], *b* [84], *c* [81], *d* [98], *e* [42], *f* [99]). *Green* indicates NPC1L1 disruption by ezetimibe or genetic deletion (α and γ [100], β [101]). *Blue symbols*: an LXR agonist (i [102]), ABCG5/G8 knock-in (iv [103]), atrovastatin (ii [84]), or apical sodium codependent bile acid transporter inhibition (iii [104], v [105])

Phytosterol-supplemented diets reduce circulating cholesterol levels and atherosclerotic lesions in hamsters [97] and genetically modified hyperlipidemic mice (*Apoe*^−/−^ or *Ldlr*^−/−^) (Fig. 5). Thus, phytosterol-supplemented diets appear to be beneficial, at least in rodents, with the effects comparable to other approaches that attenuate the development of atherosclerosis.

### Known Atherogenic Factors that Cause Concomitant Increases in Phytosterol Levels

#### In Patients with Sitosterolemia

**Fig. 6:**
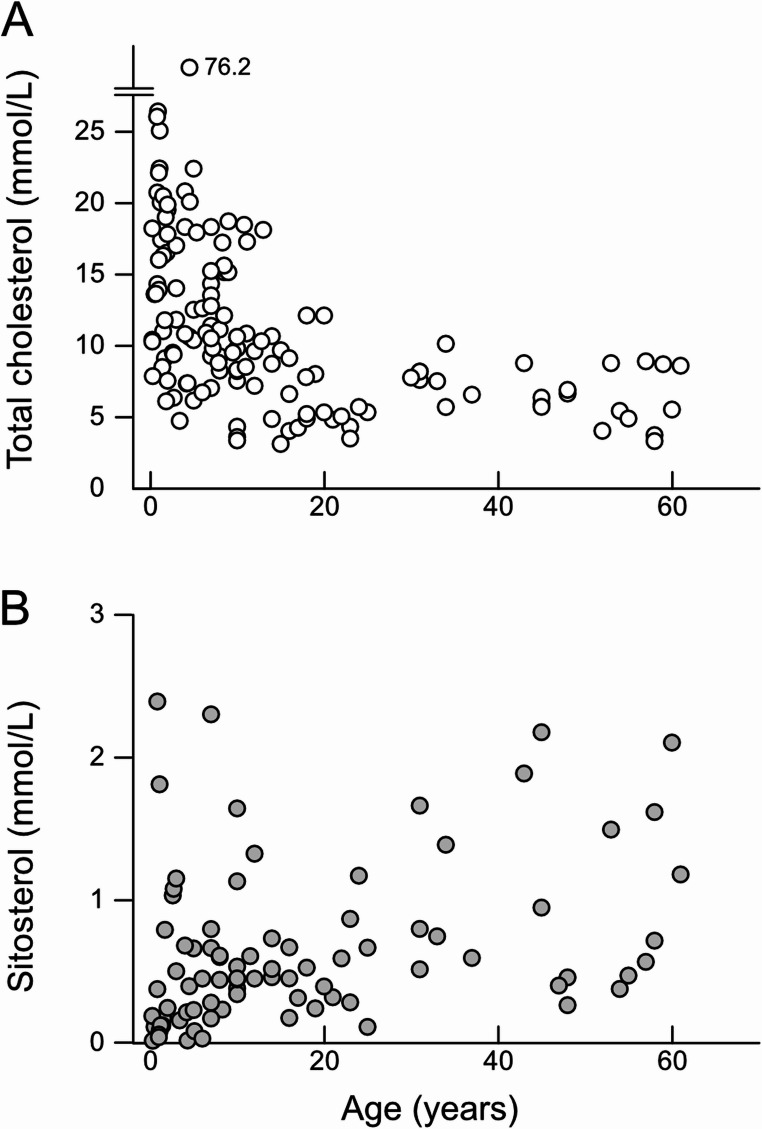
Age-dependent severe hypercholesterolemia in patients with sitosterolemia. Circulating cholesterol (***A***) and sitosterol (***B***) levels in patients with sitosterolemia at pretreatment. For data used in these plots, see Supplementary Table 1

Windler et al. [95] showed that children who suffered from sitosterolemia also had severe hypercholesterolemia (*n* = 4, > 13 mmol/L total cholesterol). Hansel et al. [92] argued that severe hypercholesterolemia is a transient phenomenon frequently observed in the patients. This suggests that the burden of hypercholesterolemia is most pronounced at early stages of the condition. We conducted a meta-analysis of circulating cholesterol and sitosterol levels in patients with sitosterolemia (For the detailed procedure, see Supplementary information; the data used in Fig. 6 are available in Supplementary Table 1).

Results of the analysis showed a trend that severe hypercholesterolemia occurs in the first decade of life and then cholesterol levels decline rapidly in adulthood (Fig. 6A). No such declines were observed in phytosterol levels (Fig. 6B). Thus, the early exposure of the circulatory system to excess cholesterol may be underestimated while linking sitosterolemia to accelerated atherosclerosis [95].

The exact mechanisms underlying severe childhood hypercholesterolemia remain unknown. While increased cholesterol absorption in sitosterolemic patients has been proposed as a causative factor, in practice, only about a 10% increase in absorption efficiency has been observed in adult patients, suggesting this may not fully account for the condition [33, 106]. Even if cholesterol is absorbed in excess, it should be balanced with increased cholesterol excretion and reduced *de novo* cholesterol synthesis (Fig. 4A). The gradual reduction in circulating cholesterol levels over the first 10 years of life indicates epigenetic adaptations for cholesterol homeostasis in these patients. Gene expression of cytochrome P450 enzymes is particularly increased in the adult livers compared to fetal livers [107]. These enzymes are associated with cholesterol homeostasis via generating LXR ligand oxysterols and stimulating bile acid synthesis [108, 109]. In adult patients with sitosterolemia, cholesterol synthesis was attenuated to less than a half of normal levels [106]. Such a feedback system may be immature in infants or disrupted by increased phytosterols [87].

Breastfeeding may be partly responsible for such hypercholesterolemic conditions, as breast milk contains approximately 150 mg/dL (~ 4 mmol/L) of cholesterol [86]. There have been two cases with sitosterolemia whose cholesterol levels were normalized after stopping breastfeeding (19 mmol/L to 5 mmol/L [110];17 mmol/L to 5 mmol/L [111]). Note that, in healthy infants, breastfeeding increases plasma cholesterol levels by only approximately 10% [112].

It is uncertain whether this early cholesterol exposure is common in all patients with sitosterolemia. Not only is the disease extremely rare, but also many cases with sitosterolemia may remain undiagnosed [113] or often be misdiagnosed as familial hypercholesterolemia [114]. Furthermore, definitive diagnosis is likely to be biased toward patients with obvious manifestations, such as those presenting with xanthoma or simply family members of probands [115]. Therefore, the meta-analysis shown in Fig. 6 might be biased toward the phenotype associated with hypercholesterolemia.

Furthermore, the fact that premature atherosclerosis occurs in some, but not all, patients with sitosterolemia—including those with hypercholesterolemia and even among affected siblings—suggests that factors other than elevated cholesterol, such as the genetic background, may play a significant role in the pathogenesis of premature atherosclerosis [92, 93].

#### In Individuals with Normo-Phytosterolemia

A subgroup analysis of the Scandinavian Simvastatin Survival Study (4S) showed that individuals in the highest quartile of cholesterol absorption had a 2.2-fold increase in recurrent coronary events compared with those in the lowest quartile [116, 117]. Since the rate of phytosterol absorption positively correlates with that of cholesterol absorption, these individuals may also have increased circulating phytosterols. In addition, patients with established cardiovascular disease in the Framingham Offspring Study Cycle-6 exhibit higher phytosterol levels than matched controls [118]. Other studies reported that the severity or the incidence of coronary heart disease was positively associated with phytosterol levels [119, 120]. These findings suggest that it is difficult to distinguish the impact of phytosterols from that of increased cholesterol absorption on the risk assessment of cardiovascular diseases.

Mendelian randomization studies have shown that circulating phytosterol levels are associated with an increased risk of cardiovascular events [25, 26]. However, as stated above, circulating phytosterol levels are closely linked to cholesterol absorption efficiency, an epidemiologically implicated cardiovascular risk factor [116, 118, 121]. The exclusion restriction assumption—an essential requirement for Mendelian randomization—states that the genetic instrument should influence the outcome only through the exposure of interest and not via other pathways. Thus, the exclusion restriction was violated in these studies and the genetic instrument could affect the outcome through other than the exposure pathways [122].

### Synthesis of Evidence: Benefits and Risks of Dietary Phytosterol Intake

Sitosterolemic animal models exhibit a range of organ dysfunctions probably due to phytosterol deposition similar to those observed in patients with sitosterolemia, yet have not provided evidence supporting the atherogenic potential of phytosterols. In rodents with functionally intact *Abcg5/g8*, phytosterol-supplemented diets reduced atherosclerotic lesions. In humans, consumption of phytosterol-supplemented foods lowers circulating cholesterol levels by approximately 10% without substantially increasing circulating phytosterol concentrations. While Mendelian randomization studies have raised concerns about phytosterol intake, these investigations may have assessed the risks associated with confounding factors rather than phytosterols per se. Importantly, these studies were not specifically designed to evaluate the risks of dietary phytosterol consumption. Based on this evidence, dietary phytosterol intake likely confers cardiovascular benefits by reducing ASCVD risk**—**unless the minimal elevation in circulating phytosterols during the intake outweighs the protective effects expected from a 10% reduction in circulating cholesterol levels.

Although public health recommendations advocating dietary phytosterol intake raise theoretical concerns about the risk of inadvertent intake by patients with sitosterolemia, evidence from case reports suggests that such consumption may not cause additional elevation of their already elevated circulating phytosterol levels [123].

## Conclusions

In this review, we presented data on circulating phytosterol levels in patients with sitosterolemia, individuals with *normo-phytosterolemia*, and animal models. The consequences of *hyper-phytosterolemic* conditions differ markedly from those of *normo-phytosterolemia*; thus, these two states should be considered separately.

While *hyper-phytosterolemic* conditions impair cellular and tissue function due to sterol deposition, they are unlikely to directly induce premature atherosclerosis. Instead, early-life exposure to hypercholesterolemic conditions in patients with sitosterolemia may be responsible for it.

Phytosterols are nutritionally unnecessary for humans, and ABCG5/G8 maintains their circulating levels at a low threshold even when increased phytosterol intake. Meanwhile, phytosterol-supplemented foods can lower circulating cholesterol by approximately 10% via both inhibiting cholesterol absorption and increasing fecal neutral sterol excretion [13, 16].

A meta-analysis by Law et al. [124] demonstrated that 10% reduction in circulating cholesterol in individuals in their forties can ultimately reduce ASCVD incident by half. The Pathobiological Determinants of Atherosclerosis in Youth (PDAY) Study revealed that atherosclerosis begins in youth, prompting the authors to advocate for primary prevention starting in childhood or adolescence [125]. Consequently, increasing the intake of phytosterol-supplemented foods may help reduce ASCVD risk not only in individuals seeking to improve lipid control, but also in young, risk-conscious individuals.

## Key References

- Helgadottir A, Thorleifsson G, Alexandersson KF, Tragante V, Thorsteinsdottir M, Eiriksson FF, et al. Genetic variability in the absorption of dietary sterols affects the risk of coronary artery disease. Eur Heart J. 2020;41(28):2618-28.10.1093/eurheartj/ehaa531.
  - This Mendelian randomization study found that higher circulating phytosterol levels are associated with an increased risk of coronary artery disease (CAD), supporting a cautious approach to phytosterol-supplemented diets. However, Plat et al. (Eur Heart J. 2020;42(3):281-2) raised concerns about the interpretation of these findings and highlighted the complexity of the relationship between phytosterols and CAD risk.
- Windler E, Beil F-U, Berthold HK, Gouni-Berthold I, Kassner U, Klose G, et al. Phytosterols and cardiovascular risk evaluated against the background of phytosterolemia cases**—**A German expert panel statement. Nutrients. 2023;15(4):828. 10.3390/nu15040828.
  - This review summarizes evidence indicating that the risks attributed to phytosterols have likely been overstated, and suggests that any actual risk is probably marginal, though the authors note the challenges in definitively proving this point. Our current review further strengthens these arguments by providing complementary quantitative evidence.
- Wang L, Feng L, Prabahar K, Hernández-Wolters B, Wang Z. The effect of phytosterol supplementation on lipid profile: a critical umbrella review of interventional meta-analyses. Phytother Res. 2024;38(2):507-19. 10.1002/ptr.8052.
  - The umbrella meta-analysis revealed a statistically significant LDL-C reduction (approximately a 6–8% decrease from baseline) in typical populations with phytosterol-supplemented diets. This effect size is clinically meaningful, as even modest LDL-C reductions translate to cardiovascular benefit.

## Supplementary Information

Below is the link to the electronic supplementary material.

Supplementary Material 1 (PDF 181 KB)

Supplementary Material 2 (PDF 688 KB)

## Data Availability

No datasets were generated or analysed during the current study.

## Declarations

### Figures Originality

All the figures are original and created by the authors.
