# Supplementary material for: Factors Affecting Circulating Phytosterol Levels: Toward an Integrated Understanding of Atherogenicity and Atheroprotection by Dietary and Circulating Phytosterols
Source: Curr Atheroscler Rep. 2025 Oct 21;27(1):104. doi: 10.1007/s11883-025-01334-7 (PMC12540574; doi:10.1007/s11883-025-01334-7)
Supplement: Supplementary file 1 — (PDF 181 KB) [file 11883_2025_1334_MOESM1_ESM.pdf]

August 1st 2025

Supplementary information

Data collection procedure for age-related changes of cholesterol and sitosterol levels in patients with sitosterolemia

Current Atherosclerosis Reports

Factors affecting circulating phytosterol levels: Toward an integrated understanding of atherogenicity and atheroprotection by dietary and circulating phytosterols

Takanari Nakano
Department of Biochemistry, Faculty of Medicine, Saitama Medical University, Saitama, Japan
tnakano@saitama-med.ac.jp

A literature search was conducted to identify all published cases of sitosterolemia until July 2025. The following databases were systematically searched from inception to July 27th, 2025:

PubMed/MEDLINE, Perplexity Pro (AI-powered research database), References in the book "The Metabolic and Molecular Bases of Inherited Disease, 8th edition"

No language restrictions were applied to ensure comprehensive coverage in the search. We used Perplexity Pro's artificial intelligence translation capabilities to extract relevant data from the papers. Hand-searching of reference lists from included studies and relevant review articles was also performed to identify additional cases.

Inclusion criteria:

– Case reports, case series, and observational studies describing patients with biochemically and/or genetically confirmed sitosterolemia

– Studies reporting total cholesterol and phytosterol levels at diagnosis (before treatment initiation)

– Patients of any age and ethnicity

– Studies published in any language

Exclusion criteria:

– Patients who had received treatment for hyperlipidemia prior to biochemical assessment

– Cases where medications or comorbid conditions could potentially influence total

cholesterol or phytosterol levels

– Patients with additional genetic disorders or diseases that could independently affect the measured parameters

– Review articles, editorials, and conference abstracts without original patient data

– Studies lacking sufficient clinical or biochemical data for analysis

Study Selection and data retrieval

Titles and abstracts were screened for potential relevance. Subsequently, full-text articles of potentially eligible studies were retrieved and assessed against the predefined inclusion and exclusion criteria. The age of cases, Total cholesterol and sitosterol levels were extracted from each included study. Other variables, such as LDL-C and campesterol, were also extracted if available.
