# Supplementary material for: Factors Affecting Circulating Phytosterol Levels: Toward an Integrated Understanding of Atherogenicity and Atheroprotection by Dietary and Circulating Phytosterols
Source: Curr Atheroscler Rep. 2025 Oct 21;27(1):104. doi: 10.1007/s11883-025-01334-7 (PMC12540574; doi:10.1007/s11883-025-01334-7)
Supplement: Supplementary file 2 — (PDF 688 KB) [file 11883_2025_1334_MOESM2_ESM.pdf]

Supplementary Table 1. Lipid profiles in patients with sitosterolemia at pretreatment

Current Atherosclerosis Reports

Nakano et al., Factors affecting circulating phytosterol levels: Toward an integrated understanding of atherogenicity and atheroprotection by dietary and circulating phytosterols

Department of Biochemistry, Faculty of Medicine, Saitama Medical University, Saitama, Japan
tnakano@saitama-med.ac.jp

Year	Patients	Sex	Age (y)	TC	LDL-C	HDL-C	TG	Sitosterol	Campesterol	Stigmasterol	ID
1974	LH	F	22	5.0			0.9	0.59	0.25		#5411
1974	RH	F	20	5.3			0.7	0.39	0.18	0.02	#5411
1990	2	M	55	4.9		1.4	3.6	0.47	0.29		#5714
1990	3	F	48	6.9		1.1	1.6	0.46	0.28		#5714
1990	4	F	45	6.4		2.1	0.6	0.95	0.60		#5714
1990		M	7	14.3							#5662
1992		M	33	7.5				0.74	0.51		#5887
1994	CIII3	F	8	8.2			2.4	0.60			#5897
1994	KIII2	F	10	8.3				1.13			#5897
1994	KIII3	F	5	6.1				0.66			#5897
1994	RII1		16	6.6				0.45			#5897
1997	Subject4/siser	F	8	11.1		0.7		0.61	0.49		#5898
1997	Subject5/Proband	F	6	6.7		0.8		0.45	0.35		#5898
2000	Patient 1	F	0.83	20.7		1.0	1.0	0.38	0.18	0.01	#5653
2000	Patient 2	F	0.5	13.6		1.3	1.2	0.11	0.05	0.01	#5653
2000		F	7	11.4	9.7	1.5	0.6				#5659
2002		M	7	18.3		1.3	1.2	2.30			#5607
2003		M	4	18.3	16.4	1.3	1.2				#5608
2003		M	19	8.0	6.3			0.24			#5664
2004	II-1	F	5	12.5			3.9				#5908
2004	II-2	F	4	10.8	9.0	0.7	2.2	0.68			#5908
2004	II-3	M	3	11.8	10.1	1.1	0.9	1.15			#5908
2004	II-4	F	8	9.2	7.5	0.9	2.0	0.44			#5908
2007		F	10	9.8			1.1	0.38			#5609
2010	Patient 1	F	1.5	11.0	8.9	1.7	1.7	0.17	0.09		#3070
2010	Patient 2	F	0.25	18.2	14.6						#3070
2010	Patient 3, sister of patient [illegible]	F	0.25	10.4	7.8						#3070
2010	Patient 4	F	1.9	16.5	13.4						#3070
2010	Patient 5	F	12	9.6							#3070
2010		F	0.9	26.4	21.6	1.4	1.4	0.06			#5613
2011		M	31	8.2	5.4			0.80		0.22	#5627
2012		M	47					0.40	0.20		#6020
2014	Patient 1	F	18	12.1							#2971
2014	Patient 2	F	13	18.1	15.5						#2971
2014		F	4	20.8							#2971
2014		F	9	18.7	16.3						#2971
2014		NA	10	10.6	1.0						#2971
2014		F	1.25	17.4	13.9						#5605
2014		F	25	5.3				0.67			#3115
2014		F	24	5.7				1.17			#3115
2014		M	23	3.5				0.87			#3115
2014		F	34	5.7				1.39			#3115
2014		F	43	8.8				1.89			#3115
2014		M	61	8.6				1.18			#3115
2014		M	58	3.7				0.71			#3115
2014		M	57	8.9				0.57			#3115
2014		F	53	8.8				1.49			#3115
2014		M	31	8.2				1.66			#3115
2014		M	58	3.3				1.62			#3115
2014		F	45	5.9				2.17			#3115
2014		F	60	5.5				2.10			#3115
2014		M	16	9.1	7.5	0.9	1.1				#6056
2014		F	59	8.7	6.6	1.6	1.2				#6026
2015		F	0.83	14.3				2.39			#5606
2015		F	1.08	22.4	20.5			1.81			#5606
2015		F	2	19.5	15.2			0.24			#5606

2016		F	10	7.5	5.8		2.6	1.64	0.81		#5615
2016		M	12					1.32	1.01	0.03	#6015
2016		F	8.5	15.6			1.0				#6019
2017	Case 1	F	2	19.9	17.8	1.0	2.3				#6040
2017	Case 2	M	7	13.5	11.2	1.3	0.6				#6040
2017		M	16	4.0		1.4	0.6	0.17			#5614
2017		F	5	22.4				0.23			#6042
2018	II.2	F	1	13.9	11.9	1.0	2.7	0.04			#6035
2018		M	1.7	9.1				0.79			#5629
2018		M	2.6	9.5				1.03			#5629
2018		M	2.7	9.4				1.08			#5629
2018		F	4.5	10.6				0.40			#5629
2018		F	5	10.4							#5629
2018		F	7	7.0				0.66			#5629
2018		F	7	9.3				0.80			#5629
2018		M	10	4.3				0.34			#5629
2018		M	10	3.6				0.53			#5629
2018		F	10	8.2				0.44			#5629
2018		F	12	7.2				0.45			#5629
2018		F	14	8.7				0.73			#5629
2018		M	14	4.9				0.46			#5629
2018		M	17	4.2				0.31			#5629
2018		M	18	4.9				0.53			#5629
2018		M	21	4.8				0.32			#5629
2018		F	23	4.3				0.28			#5629
2018		F	31	7.6				0.51			#5629
2018		F	37	6.6				0.59			#5629
2018		M	48	6.6				0.26			#5629
2018		F	54	5.4				0.38			#5629
2018		M	1.5	20.5	14.7						#6034
2018		F	2	17.8	14.2						#6044
2018		F	25		5.7			0.11	0.11		#6030
2019	Patient 1	F	8.5	12.1	10.2						#6012
2019		M	14	10.7	8.6			0.52	0.20	0.06	#6031
2020		F	12.8	10.3		1.9	1.3				#6037
2021		M	8.5	15.1							#5634
2021		F	10	9.8							#5888
2022	III1	M	7	15.2	13.3	1.2	1.2	0.28	0.03	0.01	#6028
2022	Index case	M	10	3.4			1.0	0.45	0.14		#6022
2022	Patient 1	M	9	15.1	14.0	2.0	2.0				#6013
2022		F	3	14.0	10.9			0.50	0.11		#5597
2022		F	3.4	4.7	3.8			0.16	0.04		#5597
2022		M	0.25	10.3	8.1			0.19	0.13		#5597
2022		F	1.4	8.5	7.6			0.12	0.06		#5597
2022		F	34	10.1							#5891
2022		F	10	10.6	6.5	1.6	0.5				#6036
2023	Case 1	F	1	16.0							#6016
2023	Case 2	F	6	12.6				0.03	0.01		#6016
2023		M	30	7.8	6.0		0.9				#5739
2023		F	20	12.1	11.1		0.7				#6052
2023		M	52	4.0	2.4	1.3	0.9				#6052
2023		M	0.66	13.6	11.6	1.0	1.8				#6046
2024	Patient 1	M	7								#5869
2024		F	66								#6011
2024		F	3	17.0	8.2	0.6	0.7				#6023
2024		M	7	12.8	11.0	1.3	1.0				#5689
2024		M	45	5.7	3.7	1.2					#6027
2025	P1	M	10.8	18.5	16.0		0.7				#6006
2025	P10	F	1	22.1	16.4		0.9	0.06	0.06	0.00	#6006
2025	P11	F	0.3	7.9	5.6		0.9	0.01	0.02	0.00	#6006
2025	P12	M	7	10.5	9.3		0.5	0.17	0.12	0.02	#6006
2025	P2 (older sister)	F	8.3	15.4	13.5		1.2	0.23	0.17	0.01	#6006
2025	P3 (younger brother)	M	4.2	7.3	5.9		1.2	0.21	0.15	0.01	#6006
2025	P4	F	1.1	25.1	17.7		0.9	0.08	0.08	0.01	#6006
2025	P5	F	0.8	26.0	20.5		1.1				#6006
2025	P6	F	4.3	7.4	4.5		0.6	0.01	0.02	0.00	#6006
2025	P7	F	1.2	20.0	15.2		1.7	0.12	0.14	0.01	#6006

2025	P8	M	5.1	76.9	74.4	1.8	0.08	0.14	0.01	#6006
2025	P9	M	7.2	9.8	8.7	1.0				#6006
2025	Patient 1	M	1.5	16.3	13.4					#6009
2025	Patient 2	M	11	8.5	4.6					#6009
2025	Patient 3	F	18	5.2	3.5					#6009
2025	Patient 4	F	15	3.1	1.9					#6009
2025	S1, Sibling of S2	F	1.68	11.8	8.4					#6005
2025	S10	M	11.14	17.3	11.7					#6005
2025	S11	F	11.08	10.8	7.0					#6005
2025	S12	F	15.02	9.7	7.5					#6005
2025	S13, Sibling of S14	F	9.51	9.5	8.1					#6005
2025	S14, Sibling of S13	F	17.93	7.8	6.3					#6005
2025	S2, Sibling of S1	M	2.72	6.3	4.4					#6005
2025	S3	M	8.31	17.2	13.5					#6005
2025	S4	M	4.56	20.1	14.7					#6005
2025	S5	M	1.84	6.1	4.7					#6005
2025	S6	M	6.44	10.9	7.8					#6005
2025	S7	F	7.86	8.8	6.2					#6005
2025	S8	M	1.78	19.0	16.8					#6005
2025	S9	F	5.35	17.9	13.0					#6005
2025	Sit 1C	M	16				0.67			#6041
2025	Sit 1F	F	11.5				0.61			#6041
2025		F	2	7.5	5.2					#6038

All lipid concentrations are expressed in mmol/L. TC, total cholesterol; TG, triglyceride.
Patient information in the "Patients" column is derived from the respective studies.

ID	References
#2971	Hansel B, Carrié A, Brun-Druc N, Leclert G, Chantepie S, Coiffard A-S, et al. Premature atherosclerosis is not systematic in phytosterolemic patients: Severe hypercholesterolemia as a confounding factor in five subjects. Atherosclerosis. 2014;234(1):162-8.
#3070	Niu D-M, Chong K-W, Hsu J-H, Wu T-T, Yu H-C, Huang C-H, et al. Clinical observations, molecular genetic analysis, and treatment of sitosterolemia in infants and children. J Inherit Metab Dis. 2010;33(4):437-43.
#3115	Wang Z, Cao L, Su Y, Wang G, Wang R, Yu Z, et al. Specific macrothrombocytopenia/hemolytic anemia associated with sitosterolemia. Am J Hematol. 2014;89(3):320-4.
#5411	Bhattacharyya AK, Connor WE. β-Sitosterolemia and xanthomatosis: A newly described lipid storage disease in two sisters. J Clin Invest. 1974;53(4):1033-43.
#5597	Zhou Z, Su X, Cai Y, Ting TH, Zhang W, Lin Y, et al. Features of chinese patients with sitosterolemia. Lipids Health Dis. 2022;21(1):11.
#5605	Park JH, Chung IH, Kim DH, Choi MH, Garg A, Yoo E-G. Sitosterolemia presenting with severe hypercholesterolemia and intertriginous xanthomas in a breastfed infant: Case report and brief review. J Clin Endocrinol Metab. 2014;99(5):1512-8.
#5606	Tada H, Kawashiri MA, Takata M, Matsunami K, Imamura A, Matsuyama M, et al. Infantile cases of sitosterolaemia with novel mutations in the ABCG5 gene: Extreme hypercholesterolaemia is exacerbated by breastfeeding. JIMD Rep. 2015;21:115-22.
#5607	Lam C-W, Cheng AW-F, Tong S-F, Chan Y-W. Novel donor splice site mutation of ABCG5 gene in sitosterolemia. Mol Genet Metab. 2002;75(2):178-80.
#5608	Cheng WF, Yuen YP, Chow CB, Au KM, Chan YW, Tam SC. Sitosterolaemia and xanthomatosis in a child. Hong Kong Med J. 2003;9(3):206-9.
#5609	Mannucci L, Guardamagna O, Bertucci P, Pisciotta L, Liberatoscioli L, Bertolini S, et al. β-sitosterolaemia: a new nonsense mutation in the ABCG5 gene. Eur J Clin Invest. 2007;37(12):997-1000.
#5613	Rios J, Stein E, Shendure J, Hobbs HH, Cohen JC. Identification by whole-genome resequencing of gene defect responsible for severe hypercholesterolemia. Hum Mol Genet. 2010;19(22):4313-8.
#5614	Nagahiko S, Hayato T. Lipoprotein apheresis for sitosterolemia. Ann Intern Med. 2017;167(12):896-9.
#5615	Colima Fausto AG, González García JR, Wong Ley Madero LE, Magaña Torres MT. Two novel mutations in the ABCG5 gene, c.144 -1G>A and c.1523 delC, in a Mexican family with sitosterolemia. J Clin Lipidol. 2016;10(1):204-8.
#5627	Wang G, Wang Z, Liang J, Cao L, Bai X, Ruan C. A phytosterolemia patient presenting exclusively with macrothrombocytopenia and stomatocytic hemolysis. Acta Haematol. 2011;126(2):95-8.
#5629	Mymin D, Salen G, Triggs-Raine B, Waggoner DJ, Dembinski T, Hatch GM. The natural history of phytosterolemia: Observations on its homeostasis. Atherosclerosis. 2018;269:122-8.
#5634	Ba H, Peng H, He X, Cheng L, Lin Y, Li X, et al. Sitosterolemia with atherosclerosis in a child: A case report. Front Pediatr. 2021;9
#5653	Yoshida A, Naito M, Miyazaki K. Japanese sisters associated with sseudohomozygous familial hypercholesterolemia and sitosterolemia. J Atheroscler Thromb. 2000;7(1):33-8.
#5659	Alam M, Garzon MC, Salen G, Starc TJ. Tuberous xanthomas in sitosterolemia. Pediatr Dermatol. 2000;17(6):447-9.
#5662	Belamarich PF, Deckelbaum RJ, Starc TJ, Dobrin BE, Tint GS, Salen G. Response to diet and cholestyramine in a patient with sitosterolemia. Pediatrics. 1990;86(6):977-81.
#5664	Katayama S, Satoh T, Yagi T, Hirose N, Kurita Y, Anzai T, et al. A 19-year-old man with myocardial infarction and sitosterolemia. Int Med. 2003;42(7):591-4.
#5689	Rees DC, Iolascon A, Carella M, O'Marcaigh AS, Kendra JR, Jowitt SN, et al. Stomatocytic haemolysis and macrothrombocytopenia (Mediterranean stomatocytosis/macrothrombocytopenia) is the haematological presentation of phytosterolaemia. British Journal of Haematology. 2005;130(2):297-309.
#5714	Hidaka H, Nakamura T, Aoki T, Kojima H, Nakajima Y, Kosugi K, et al. Increased plasma plant sterol levels in heterozygotes with sitosterolemia and xanthomatosis. J Lipid Res. 1990;31(5):881-8.
#5739	Mahzari MM. Sitosterolemia: A case report and a concise literature review. Case Rep Endocrinol. 2023;2023:4451595.
#5869	Hernández-Mijares A, Bañuls C, Jover A, Solá E, Bellod L, Martínez-Triguero ML, et al. Low intestinal cholesterol absorption is associated with a reduced efficacy of phytosterol esters as hypolipemic agents in patients with metabolic syndrome. Clin Nutr. 2011;30(5):604-9.
#5887	Watts GF, Mitchell WD. Clinical and metabolic findings in a patient with phytosterolaemia. Ann Clin Biochem. 1992;29(2):231-6.
#5888	Frederiksen TC, Mortensen MB, Kanstrup HL. Seventeen years of misdiagnosis in rare dyslipidaemia: a case report of sitosterolaemia in a young female. Eur Heart J Case Rep. 2021;5(5):ytab188.
#5891	Limonova AS, Ershova AI, Meshkov AN, Kiseleva AV, Divashuk MG, Kurkina MV, et al. Case Report: Next Generation Sequencing in Clinical Practice–A Real Tool for Ending the Protracted Diagnostic Odyssey. Frontiers in Cardiovascular Medicine. 2022;8
#5897	Berger GMB, Deppe WM, Marais AD, Biggs M. Phytosterolaemia in three unrelated South African families. Postgrad Med J. 1994;70(827):631-7.
#5898	Hidaka H, Sugiura H, Nakamura T-A, Kojima H, Fujita M, Sugie N, et al. β-sitosterolemia with generalized eruptive xanthomatosis. Endocr J. 1997;44(1):59-64.
#5908	Wang J, Joy T, Mymin D, Frohlich J, Hegele RA. Phenotypic heterogeneity of sitosterolemia. J lipid Res. 2004;45(12):2361-7.
#6005	Do TTM, Vu CD, Dien TM, Can TBN, Nguyen TTN, Nguyen HH, et al. Phenotypes, Genotypes, Treatment, and Outcomes of 14 Children with Sitosterolemia at Vietnam National Children’s Hospital. Journal of Clinical Medicine. 2025;14(2):325.
#6006	Huang S, Du M, Wang Xo, Liu Y, Song F. Clinical, genetic characteristics and long-term follow-up of sitosterolemia in children. Translational Pediatrics. 2025;14(2):222-30.
#6009	Gamage D, Chisholm K, Kilo T, Ooi SE, Srinivasan S. Sitosterolemia—An Underdiagnosed and Heterogeneous Lipid Disorder. A Case Series From a Tertiary Care Centre in Australia. J Paediatr Child Health. 2025;61(4):635-8.
#6011	Li F, Xie X, Xu S, Zhou F, Yu Y, Fang X, et al. Cerebral involvement in sitosterolemia. Lipids in Health and Disease. 2024;23(1):222.
#6012	Veit L, Allegri Machado G, Bürer C, Speer O, Häberle J. Sitosterolemia—10 years observation in two sisters. JIMD Reports. 2019;48(1):4-10.
#6013	Su S-Q, Xiong D-S, Ding X-M, Kuang J-A, Lin Y-C. Pediatric patients with familially inherited sitosterolemia: Two case reports. Frontiers in Cardiovascular Medicine. 2022;Volume 9 - 2022
#6015	Bain BJ, Chakravorty S. Phytosterolemia. American Journal of Hematology. 2016;91(6):643-.
#6016	Miroshnikova VV, Vasiluev PA, Linkova SV, Soloviov VM, Ivanova ON, Tolmacheva ER, et al. Pediatric Patients with Sitosterolemia: Next-Generation Sequencing and Biochemical Examination in Clinical Practice. Journal of Personalized Medicine. 2023;13(10):1492.
#6019	Renner C, Connor WE, Steiner RD. Sitosterolemia Presenting as Pseudohomozygous Familial Hypercholesterolemia. Clin Med Res. 2016;14(2):103-8.
#6020	Neff AT. Sitosterolemia's stomatocytosis and macrothrombocytopenia. Blood. 2012;120(22):4283.
#6022	Gok V, Tada H, Ensar Dogan M, Alakus Sari U, Aslan K, Ozcan A, et al. A teenager boy with a novel variant of Sitosterolemia presented with pancytopenia. Clinica Chimica Acta. 2022;529:61-6.
#6023	Alquraishi AS, Rayees S. Sitosterolemia With Two Heterozygous Variants Including a Novel Mutation c.1800T>A in the ABCG5 Gene: A Case Report of a Rare Condition in a Young Saudi Girl. Cureus. 2024;16(6):e63088.
#6026	Melenotte C, Carrié A, Serratrice J, Weiller P-J. Sitosterolemia: A new mutation in a Mediterranean patient. Journal of Clinical Lipidology. 2014;8(4):451-4.
#6027	Jurado Tapiador R, González P, Hernandez-Rodriguez I. Late diagnosis of sitosterolemia in an adult case with unexplained hemolytic anemia. Int J Lab Hematol. 2024;46(6):985-7.
#6028	Shen M-f, Hu Y-n, Chen W-x, Liao L-s, Wu M, Wu Q-y, et al. Clinical and Genetic Analysis of a Family With Sitosterolemia Caused by a Novel ATP-Binding Cassette Subfamily G Member 5 Compound Heterozygous Mutation. Frontiers in Cardiovascular Medicine. 2022;Volume 9 - 2022
#6030	Kawamura R, Saiki H, Tada H, Hata A. Acute myocardial infarction in a 25-year-old woman with sitosterolemia. Journal of Clinical Lipidology. 2018;12(1):246-9.
#6031	Wang Y, Guo Y-L, Dong Q-T, Li J-J. Severe aortic valve stenosis in a 14-year-old boy with sitosterolemia. Journal of Clinical Lipidology. 2019;13(1):49-53.
#6034	Wang W, Jiang L, Chen P-P, Wu Y, Su P-Y, Wang L-Y. A case of sitosterolemia misdiagnosed as familial hypercholesterolemia: A 4-year follow-up. Journal of Clinical Lipidology. 2018;12(1):236-9.
#6035	Tada H, Nomura A, Yamagishi M, Kawashiri M-a. First case of sitosterolemia caused by double heterozygous mutations in ABCG5 and ABCG8 genes. Journal of Clinical Lipidology. 2018;12(5):1164-8.e4.
#6036	Garrido MC, Torres MP, Lorenzo LG, Gil M, Milla CP, Rodriguez-Peralto JL, et al. Foamy Cell Histiocytosis Is a Diagnostic Pitfall: A Case Report of Xanthomatosis Secondary to Sitosterolemia Mimicking Progressive Nodular Histiocytosis. The American Journal of Dermatopathology. 2022;44(10):753-5.
#6037	Kiss S, Lee JY, Pitt J, MacGregor D, Wallace J, Marty M, et al. Dig deeper when it does not make sense: Juvenile xanthomas due to sitosterolemia. JIMD Reports. 2020;56(1):34-9.
#6038	Yoldaş Çelik M, Köşeci B, Burgaç E, Yararbaş K. Reassessing very long chain fatty acids elevations: Sitosterolemia as a non-peroxisomal cause. Molecular Genetics and Metabolism Reports. 2025;42:101178.
#6040	Buonuomo PS, Iughetti L, Pisciotta L, Rabacchi C, Papadia F, Bruzzi P, et al. Timely diagnosis of sitosterolemia by next generation sequencing in two children with severe hypercholesterolemia. Atherosclerosis. 2017;262:71-7.
#6041	Naeem WB, Khan MA, Akram Z, Afridi TUK, Khattak TA, Khan MA, et al. Identification of a homozygous variant in ABCG5 by panel sequencing in a Pakistani family with sitosterolemia: Genotype-phenotype correlation and management considerations. Journal of Clinical Lipidology. 2025;19(1):156-61.
#6042	Yagasaki H, Nakane T, Toda T, Kobayashi K, Aoyama K, Ichikawa T, et al. Carotid intima media thickness in a girl with sitosterolemia carrying a homozygous mutation in the ABCG5 gene. J Pediatr Endocrinol Metab. 2017;30(9):1007-11.
#6044	Martin JM, Cuesta A, Velasco R, Herrero A, Ramon D, Monteagudo C. Two-year-old girl with tuberous xanthomas. Journal of Clinical Pathology. 2018;71(10):860-2.
#6046	Yoshida A, Aoyama K, Yamaguchi N, Suzuki A, Mizuno H, Tada H, et al. An infant with a heterozygous variant of <i>ABCG5</i> presented with hypercholesterolemia only during breastfeeding. Clinical Pediatric Endocrinology. 2023;32(2):114-8.
#6052	Nakadoi T, Katsuyama E, Matsumoto K, Shidahara K, Hirose K, Nawachi S, et al. A case of sitosterolaemia-caused systemic large-vessel stenosis mimicking Takayasu arteritis in which FDG-PET provided a clue for the differential diagnosis. Rheumatology Advances in Practice. 2023;7(3)
#6056	Webb TN, Ramratnam M, Evans RW, Orchard T, Pacella J, Erkan E. Atherosclerotic renal artery stenosis as a cause for hypertension in an adolescent patient. Pediatric Nephrology. 2014;29(8):1457-60.

*ID, Endnote reference number in the corresponding author's database
